# The scaling relationship between leaf nitrogen and phosphorus concentrations in vascular epiphytes

**DOI:** 10.3389/fpls.2025.1712082

**Published:** 2026-01-02

**Authors:** T. Hu, T. T. Zhang, D. D. Tang, S. Liu, S. Li, X. W. Hu, Y. X. Mo, W. Y. Liu

**Affiliations:** 1 Jiangxi Provincial Key Laboratory of Carbon Neutrality and Ecosystem Carbon Sink, Lushan Botanical Garden, Jiangxi Province and Chinese Academy of Sciences, Jiujiang, China; 2 Henan University of Urban Construction, Pingdingshan, China; 3 Qianxinan State Nanpanjiang Forest Farm, Xingyi, China; 4 College of Life Sciences, Anhui Normal University, Wuhu, China; 5 Yunnan Key Laboratory of Forest Ecosystem Stability and Global Change, Xishuangbanna Tropical Botanical Garden, Chinese Academy of Sciences, Mengla, China; 6 National Field Scientific Observation and Research Station of Subtropical Forest Ecosystem in Ailao Mountain, Jingdong, China

**Keywords:** nitrogen, phosphorus, scaling exponent, vascular epiphytes, epiphytic habitat

## Abstract

**Introduction:**

The scaling relationship between leaf nitrogen (N) and phosphorus (P) concentrations reflects plant adaptation strategies and evolutionary dynamics. While extensively studied in terrestrial plants, vascular epiphytes—a key yet understudied component of global biodiversity—remain poorly understood.

**Methods:**

We compiled leaf N and P data from 38 epiphyte species across tropical seasonal rainforests and subtropical montane forests in China, supplemented by a global literature synthesis. Standardized major axis (SMA) regression analyzed N-P scaling exponents (β) across forest types, functional groups, and habitats.

**Results:**

Epiphytes exhibited a distinct global N-P scaling exponent (β=0.78), significantly higher than terrestrial plants. Facultative epiphytes showed lower β in epiphytic (0.69) versus terrestrial habitats (0.91). No significant variation occurred among functional groups or forest types, suggesting conserved nutrient allocation strategies.

**Discussion:**

The elevated β underscores epiphytes’ reliance on atmospheric nutrient inputs and adaptive P retention. Habitat-driven differences highlight niche specialization, while functional group uniformity reflects stoichiometric constraints of canopy living. These findings redefine epiphyte nutrient ecology within broader plant stoichiometry theory.

## Introduction

The scaling relationship between leaf nitrogen (N) and phosphorus (P) concentrations provides an essential framework for understanding plant nutrient allocation and physiological adaptation. Because N and P are key components of proteins and rRNA, their stoichiometric balance reflects fundamental constraints on plant growth and metabolism (Niklas et al., 2005; Rivas-Ubach et al., 2012). This relationship is often described by the power function [N] = α[P]^β^, where β represents the scaling exponent (Niklas et al., 2005). Unlike a static N:P ratio, which has been widely used to indicate nutrient limitation but lacks sensitivity to variation across scales (Güsewell, 2004), β quantifies the proportional accumulation of N relative to P across species and environments. Variation in β reveals differences in nutrient investment and adaptive strategies among plant groups (Tian et al., 2018; Guo et al., 2020; Ouyang et al., 2024; Rao et al., 2025), thereby offering a mechanistic perspective on nutrient coupling and stoichiometric evolution in plant.

Generally, β is less than 1, indicating that plants require greater P-rich rRNA to sustain rapid protein synthesis (Sterner and Elser, 2002; Elser et al., 2010). Reich et al. (2010) proposed a general β ≈ 2/3 for terrestrial leaves, yet subsequent studies have revealed substantial variability, driven by both biotic and abiotic factors. Among biological drivers, plant functional types and growth strategies exert a major influence: fast-growing or deciduous species often exhibit lower β values due to their higher P demand and shorter nutrient turnover time, whereas evergreen or stress-tolerant species display higher β values reflecting P-use conservation (Tian et al., 2018; Guo et al., 2020; Rao et al., 2025). Environmental factors such as temperature, precipitation, light intensity, and soil nutrient status also play decisive roles. Warm, moist, and nutrient-rich environments typically favor lower β values, consistent with accelerated P uptake and high growth rates (Guo et al., 2020; 2021), whereas nutrient-poor or P-limited soils promote higher β values, indicating enhanced P investment efficiency and resistance to P limitation (Guo et al., 2021). Additionally, soil N–P allometry can constrain leaf nutrient coupling, implying that leaf-level β partly mirrors the stoichiometric structure of its edaphic environment (Guo et al., 2021). Together, these findings suggest that β is shaped by the interplay of biological traits, resource availability, and environmental filtering. As such, β serves not only as an indicator of nutrient allocation flexibility but also as a functional trait reflecting plant adaptation and evolutionary responses to ecological niches (Tian et al., 2018; Guo et al., 2020; Rao et al., 2025).

Most studies on leaf N-P scaling relationships have focused on terrestrial plants (Reich et al., 2010; Tian et al., 2018; Guo et al., 2021), while vascular epiphytes—plants that depend on other plants for structural support and account for approximately 10% of global plant biodiversity (Zotz et al., 2021b) —remain underexplored in this context. Unlike terrestrial plants, epiphytes lack a root system connected to the soil and do not directly extract nutrients from the soil (Zotz and Hietz, 2001; Zotz, 2016). As a result, their nutrient uptake and allocation strategies may differ significantly. Several studies have highlighted differences in leaf nutrient concentrations between epiphytes and terrestrial plants (Cardelús and Mack, 2010; Wu et al., 2020; Hietz et al., 2022; Sulaiman et al., 2023). For example, a recent study found that LNC and N:P ratios are significantly lower in epiphytes compared to terrestrial plants, while LPC does not exhibit such difference (Hietz et al., 2022). Additionally, epiphytes are expected to have lower relative growth rates than terrestrial plants (Zotz, 2016; Hietz et al., 2022). These observations suggest that β of epiphytes may differ markedly from that of terrestrial plants.

Variation in β has been documented for terrestrial plants (Tian et al., 2018; Guo et al., 2020, 2021), with its dependence on species functional groups, ecoregions and sampling sites (Tian et al., 2018). For epiphytes, significant differences in nutrient status have been observed between epiphytic ferns and seed plants. Specifically, epiphytic ferns generally exhibit higher LNC and LPC compared to seed plants (Cardelús and Mack, 2010; Hietz et al., 2022), potentially leading to differences in β between these two groups. Beyond nutrient status and canopy microclimate, additional physiological mechanisms may also influence the N–P scaling exponent (β). For example, variations in photosynthetic pathways (e.g., C_3_, C_4_, and CAM (Crassulacean Acid Metabolism) metabolism) can alter N allocation to photosynthetic enzymes and phosphorus investment in energy metabolism, thereby modulating the proportional accumulation of N and P (Elser et al., 2010; de Paula Oliveira et al., 2021). Similarly, hydraulic traits such as xylem conductivity, leaf water potential, and water-use efficiency affect nutrient transport and metabolic rates under different water availabilities, potentially influencing the N–P coupling relationship (Yuan and Chen, 2015; Li et al., 2025). In epiphytes, where water and nutrient acquisition largely depend on atmospheric inputs (Hietz et al., 2002), these physiological traits may play an even greater role in shaping β by mediating trade-offs between photosynthetic efficiency, nutrient conservation, and drought tolerance (Querejeta et al., 2018; Gotsch et al., 2022). Vascular epiphytes communities also experience high species turnover at small spatial scales (Zhao et al., 2015; Amici et al., 2020), and the canopy microclimate plays a significantly role in modulating the physiological processes of individual epiphytes (Petter et al., 2016). These ecological and environmental variations suggest that β values in epiphytes may differ across groups, sites and habitats.

In this study, we aim to address these gaps in knowledge by presenting a comprehensive analysis of leaf N-P scaling relationships in vascular epiphytes. Our objectives are twofold: First, we compare the β values of epiphytes to those of terrestrial plants, testing whether the observed differences in nutrient status and growth rates translate into significant variation in β. Second, we examine β variation across different functional groups and spatial scales to determine if it correlates with functional groups, forest types, and habitats. By integrating global data on leaf N and P concentrations with detailed local measurements from epiphytes in a subtropical moist forest and a tropical seasonal rainforest, this study offers a novel perspective on leaf N-P scaling relationships in epiphytes and contributes to understanding their adaptive strategies in diverse ecological contexts.

## Methods

### Study site

This study was conducted in the subtropical montane moist forest of Xujiaba, located in the core area of the Ailao Mountains National Natural Reserve (101°01′E, 24°32′N, 2000~2650m altitude) in Yunnan, Southwest (SW) China. The region has a subtropical monsoon climate influenced by both southwest and southeast monsoons (Liu et al., 2003). Meteorological data (2011-2019) from the subtropical Forest Ecosystem Research Station at Xujiaba (2450m) indicate an annual mean precipitation of 1730 mm, with 85% occurring between May and October, and an annual mean temperature of 11.3°C (Gnanamoorthy et al., 2021). Primary forests dominate the area (77.9% of 667km^2^) is dominated by primary forests, primarily composed of subtropical evergreen broadleaved species such as *Lithocarpus hancei, Castanopsis wattii*, and *Lithocarpus xylocarpus* (Li et al., 2013; Wen et al., 2018). These primary forests (trees>3.5cm DBH) have a basal area of 77.31 m^2^·ha^-1^, canopy openness of 5.8%, mean DBH of 19.23 cm, and tree density of 1656 trees·ha^-1^ (Li et al., 2013).Secondary forests (~16% of the total area) have regenerated for 50–100 years after disturbance and show smaller basal area (55.17 m²·ha^-^¹), lower mean DBH (9.84 cm), higher canopy openness (31%), and greater tree density (5903.33 trees·ha^-1^; Li et al., 2013). A 28.5 km^2^ reservoir adjacent to primary forests creates a humid microclimate that supports high epiphyte diversity. Facultative epiphytes capable of both terrestrial and epiphytic growth are common and provide an ideal system for N-P scaling relationships studies (Zotz, 2016; Zhang et al., 2020).

For comparison, samples were also collected from a tropical seasonal rainforest in Bubeng, within the Mengla National Natural Reserve (101°35′E, 21°37′N) in Yunnan, SW China. This region has a typical monsoon climate with a six-month dry season (November-April) and six-month rainy season (May-October), an annual mean precipitation of 1493 mm (84% in the rainy season), and an annual mean temperature of 21.8°C (Cao et al., 2006). The forest is dominated by tree species such as *Parashorea chinensis*, *Pittosporopsis kerrii*, *Garcinia cowa* and *Castanopsis echidnocarpa*.

### Dataset description

To examine variation in N and P concentrations and their scaling relationships, two datasets were compiled (Appendices A and B). The individual-level dataset (Appendix A) includes 1,803 data points for 274 species, combining: (1) 1390 individuals from 236 species (de Paula Oliveira et al., 2021; Hietz et al., 2022), and (2) original measurements from 343 individuals from 20 vascular epiphytes in Ailao Mountains and 70 individuals from 18 vascular epiphytes in Bubeng.

The species-level dataset (Appendix B) was obtained by averaging per species to reduce sampling bias and incorporatingdata from Zotz (2004) and Querejeta et al. (2018), comprising 323 species across 41 families (103 ferns, 223 seed plants).

### Vascular epiphyte sampling

In August 2018, vascular epiphytes were collected from primary forests (both interior and reservoir-adjacent) and secondary forests in Xujiaba using ladders, twig shears, and free-hand climbing. One or more conspcific individuals of small-sized epiphytes collected from the same host tree were treated as a single sample.

A total of 343 individuals from 20 species were collected (**Supplementary Table S1**): 107 individuals from reservoir-adjacent forests, 161 individuals from primary forests, and 75 individuals from secondary forests. Host tree leaves (51 individuals, 16 species) were also sampled, focusing on outer canopy layers. Epiphytic and terrestrial individuals of five facultative epiphyte species (*Briggsia longifolia, Cautleya gracilis, Chirita macrophylla, Elatostema monandrum*, and *Impatiens siculifer*) were collected from nearby trees and ground sites. All samples were washed to remove dust and mud, oven-dried at 80 °C for 48 hours, and stored in envelopes.

In July 2018, vascular epiphyte samples of the tropical seasonal rainforest were collected in Bubeng. Five 20×20 m plots were established at the lowest (709 m) and highest (869 m) altitude points of the 20-ha tropical forest dynamics plot (101°34′26″–47″E, 21°36′42″–58″N), as well as at the periphery of the 1.44-ha square plot (101°34′59″E, 21°37′2″N), with plots separated by more than 20 m. A total of 70 epiphyte individuals from 18 species and 126 tree individuals from 18 species were collected. The same processing protocols were applied.

### Chemical analysis

For each sample, leaf nitrogen concentration (LNC, in g·kg^-^¹) and leaf phosphorus concentration (LPC, in g·kg^-^¹) were measured. Samples were ground manually with a mortar to ensure complete homogenization of small leaves and then dried at 65 °C for 24 hours prior to chemical analysis. The leaf chemistry analyses were performed at the Biogeochemical Laboratory of the Xishuangbanna Tropical Botanical Garden, Chinese Academy of Sciences. LNC was determined using an elemental analyzer (Vario MAX CN, Elemental Analysensysteme GmbH, Germany). For LPC determination, 0.2–0.3 g of each sample was weighed and digested with 10 mL of HNO_3_ and 2 mL of HClO_4_. The digestion process was carried out at room temperature for 5 hours, followed by heating at 185 °C for 2 hours. After digestion, the samples were diluted to a final volume of 50 mL with deionized water. Mass-based P concentrations were quantified using an inductively coupled plasma-atomic emission spectrometer (iCAP6300, Thermo Fisher Scientific, U.S.A.).

### Data analysis

All leaf stoichiometry data were log-transformed prior to analysis due to their strongly left-skewed distributions. The N-P scaling relationships for vascular epiphytes, as well as for epiphytic ferns and seed plants across different scales, were quantified using standardized major axis (SMA) regression (Wright et al., 2005). This was implemented with the ‘sma’ function in the ‘smart’ package (Warton et al., 2012) in R. Likelihood ratio tests were employed to compare β among vascular epiphytes.

## Results

**Figure 1:**
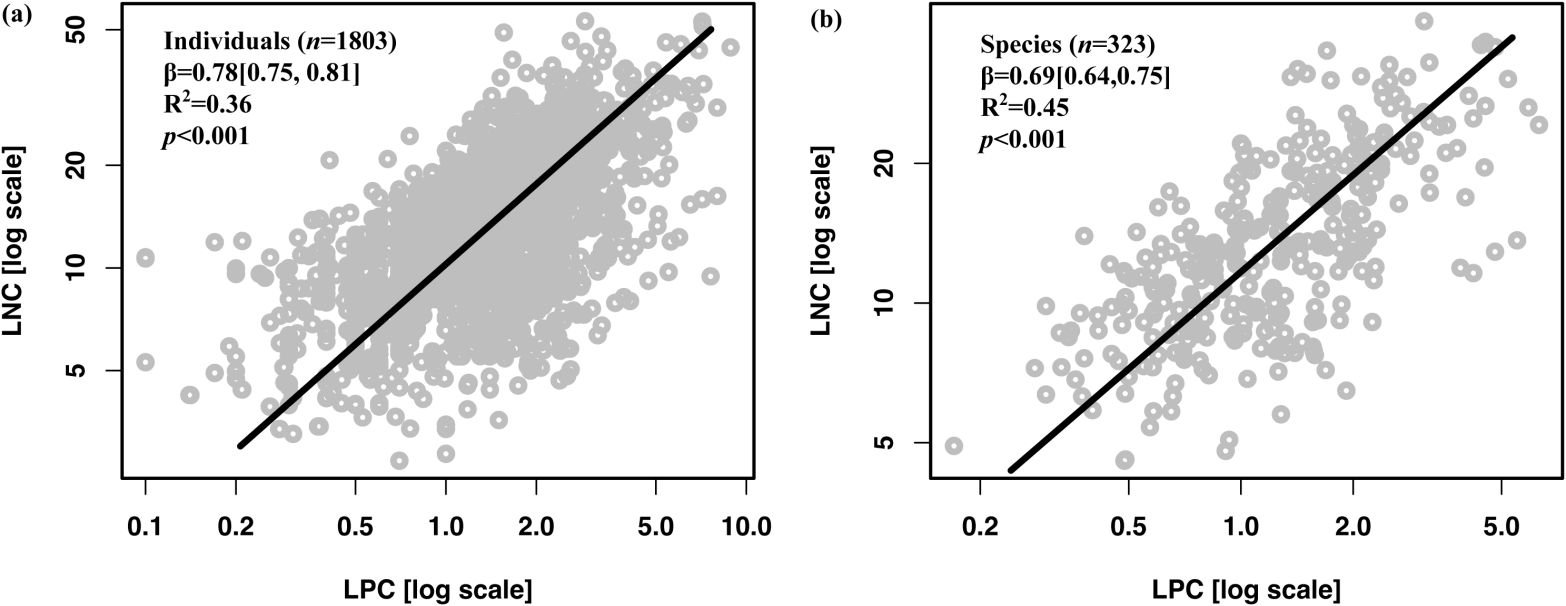
Leaf N-P scaling relationships of vascular epiphytes at global scale. **(A)** Individual level, **(B)** Species level. Scaling exponents (β) were calculated from the SMA regression between leaf N and P concentrations. The data in parentheses after the β values in the table represent the 95% confidence interval.

Global vascular epiphytes exhibited significant correlations between LNC and LPC at both individual and species levels (**Figure 1**). At the individual level, the leaf N-P scaling exponent (β) for global epiphytes was 0.78 (95% CI: 0.75–0.81), with R^2^ = 0.62 and *p* < 0.001 (**Figure 1A**). The likelihood test indicated that the exponent was significantly different from the theoretical value of 2/3 (H_0_: β = 0.667; r = 0.1931, df = 1801, *p* < 0.001). At the species level, the β was 0.69 (95% CI: 0.65–0.73), R^2^ = 0.55, *p* < 0.001 (**Figure 1B**).

**Figure 2:**
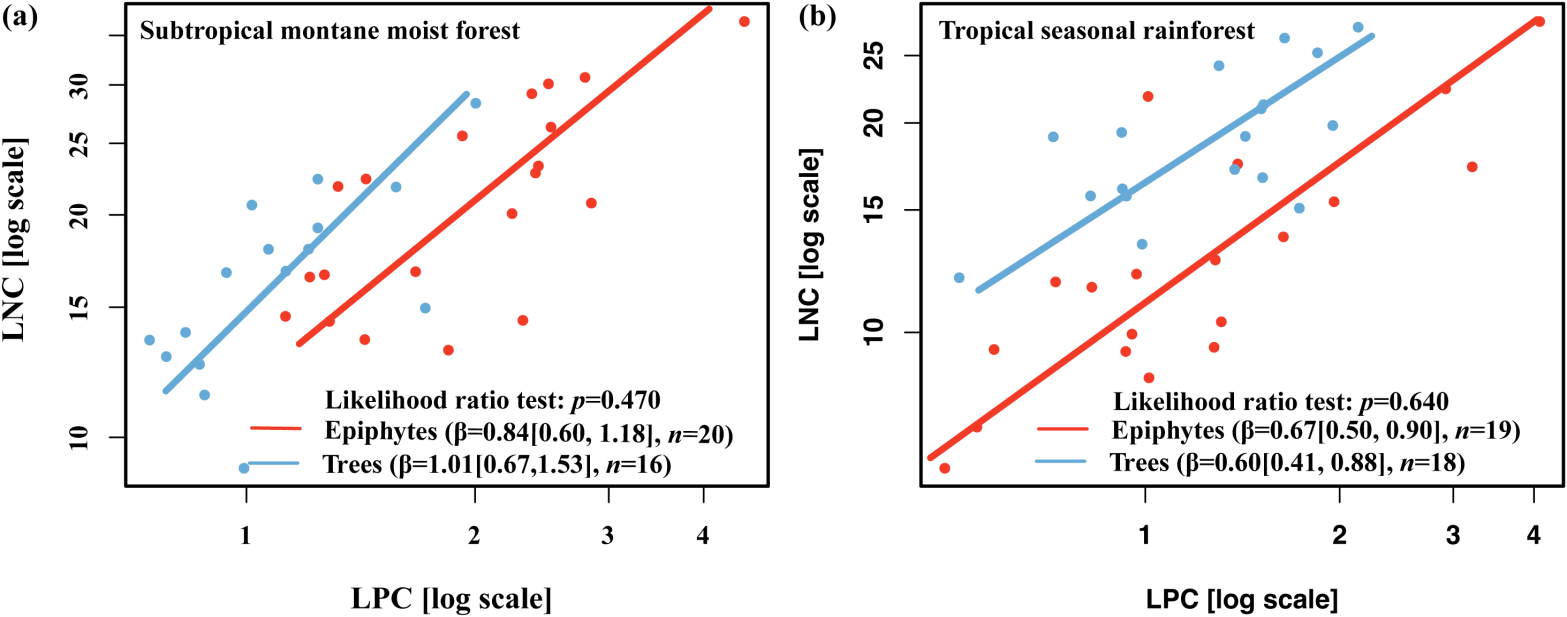
Leaf N-P scaling relationships of vascular epiphytes and trees in a subtropical montane forest **(a)** and a tropical seasonal rainforest **(b)**. The leaf N-P scaling exponents (β) were calculated from the SMA regression between leaf N and P concentrations. The data in parentheses after the β values in the table represent the 95% confidence interval. The likelihood ratio test comparing leaf N–P scaling exponents (β) among forest types for epiphytes is *p* = 0.315, and for trees is *p* = 0.061.

**Figure 3:**
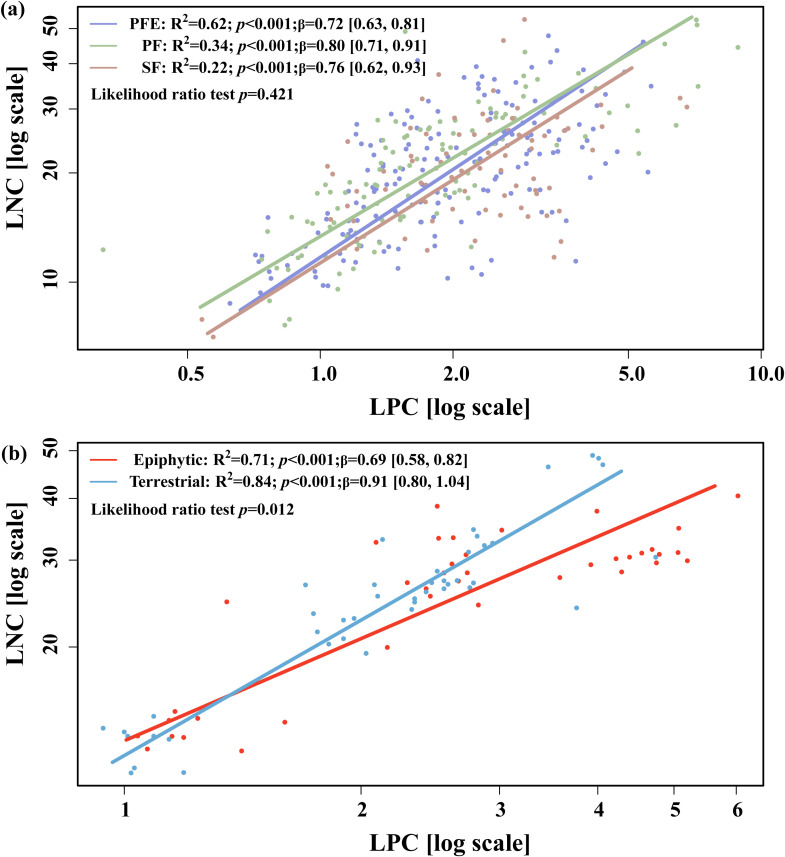
Leaf N-P scaling relationships of vascular epiphytes under different habitats at individual level **(A)**, and the epiphytic and terrestrial individuals of facultative epiphytes **(B)**. PFE, primary forest edge near the reservoir; PF, primary forest; SF, secondary forest. The N-P scaling exponents (β) were calculated from the SMA regression between leaf N and leaf P concentrations. The data in parentheses after the β values in the figure represent the 95% confidence interval.

In comparisons with trees, epiphytes in the tropical seasonal rainforest displayed significantly lower LNC (13.43 g kg^-^¹) and N:P ratio (11.05) than trees (LNC = 19.15 g kg^-^¹; N:P = 15.69), while those in the subtropical montane moist forest showed significantly higher LNC (21.47 g kg^-^¹) and leaf phosphorus concentration (LPC = 2.07 g kg^-^¹), and a lower N:P ratio (10.92) compared with trees (LNC = 16.88 g kg^-^¹; LPC = 1.14 g kg^-^¹; N:P = 15.17) (**Supplementary Table S1**). In the subtropical montane moist forest, β for vascular epiphytes was 0.84 (95% CI: 0.78–0.90; R² = 0.57; *p* < 0.001), compared to 1.01 (95% CI: 0.95–1.07; R² = 0.64; *p* < 0.001) for trees (**Figure 2A**). In the tropical seasonal rainforest, β values were 0.67 (95% CI: 0.61–0.74; R² = 0.52; *p* < 0.001) for epiphytes and 0.60 (95% CI: 0.54–0.66; R² = 0.48; *p* < 0.001) for trees (**Figure 2B**). Likelihood ratio tests confirmed that these β differences between epiphytes and trees were not statistically significant (*p* = 0.25). Facultative epiphytes exhibited significantly lower β values in epiphytic habitats (0.69 ± 0.04, R² = 0.49, *p* < 0.001) compared to terrestrial habitats (0.91 ± 0.05, R² = 0.63, *p* < 0.001; **Figure 3B**). This difference was significant (*p* = 0.012), suggesting the role of epiphytic habitats in shaping N–P scaling.

**Figure 4:**
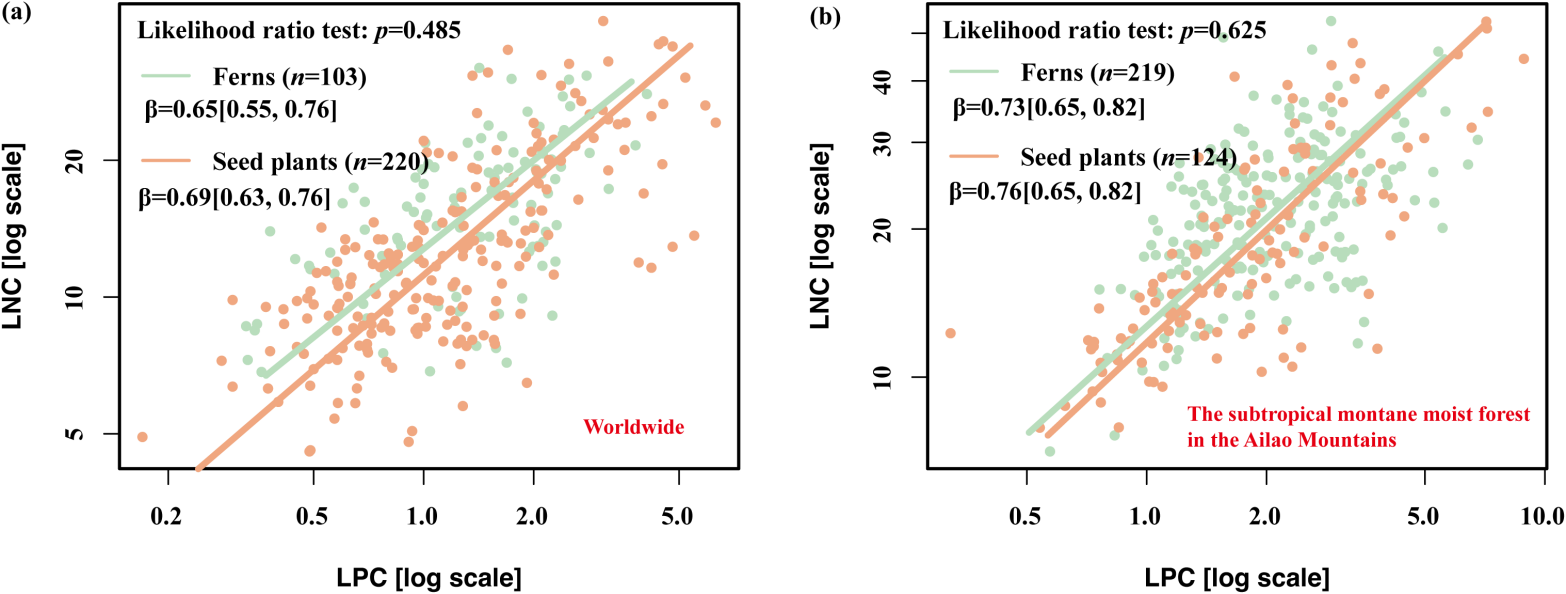
Leaf N-P scaling relationships of epiphytic ferns and seed plants at different scales. **(A)** Global scale, **(B)** Local scale. Scaling exponents (β) were calculated from the SMA regression between leaf N and P concentrations. The data in parentheses after the β values in the table represent the 95% confidence interval.

At the global scale, the mean LNC and LPC were 16.09 mg g^-1^ and 1.49 mg g^-1^ in epiphytic ferns, respectively, compared to 13.81 mg g^-1^ and 1.47 mg g^-1^ in seed plants (**Supplementary Table S1**). The corresponding N:P ratios were 13.10 and 11.75 for the two groups, respectively (**Supplementary Table S1**). Surprisingly, no significant differences in β were observed between global epiphytic ferns and seed plants (**Figure 4A**). Similarly, in the subtropical montane moist forest, β did not differ significantly between epiphytic ferns and seed plants (**Figure 4B**), despite the higher nutrient concentrations observed in ferns.

Furthermore, β showed no significant variation across forest types (*p* = 0.315, **Figure 2**) or local habitats (*p* = 0.421, **Figure 3A**). In the subtropical montane moist forest, β were 0.72 in primary forests adjacent to the reservoir, 0.80 in primary forests, and 0.76 in secondary forests (**Figure 3A**). Notably, the strength of the leaf N-P correlation declined from 0.62 (R^2^ of the SMA fit) in primary forest forests adjacent to the reservoir to 0.34 in primary forests and to 0.22 in secondary forests (**Figure 3A**), indicating a tighter coupling of LNC and LPC in habitats with more favorable growing conditions.

## Discussion

**Figure 5:**
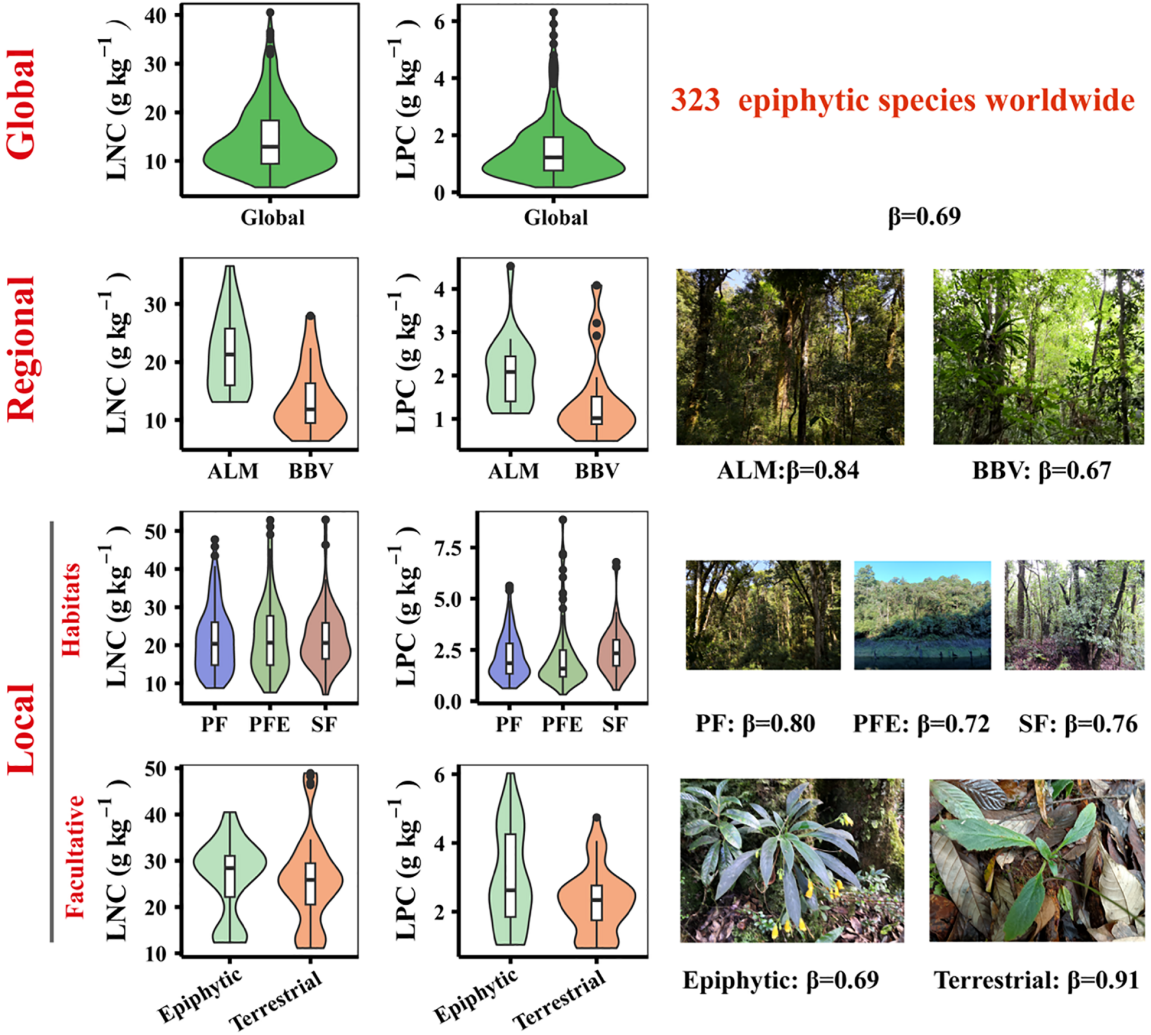
Leaf N and P concentrations and their N-P scaling exponents (β) of vascular epiphytes from global to local scales. The Leaf N-P scaling exponents (β) were calculated from the SMA regression between LNC and LPC. The global leaf β values of vascular epiphytes were calculated based on the leaf N and P contents from the global epiphyte functional traits dataset (Hietz et al., 2022), other relevant studies (Zotz, 2004; Querejeta et al., 2018; de Paula Oliveira et al., 2021) and this study. The leaf β values of vascular epiphytes at the regional and local scales were calculated based on the leaf N and P concentrations of vascular epiphytes in the subtropical montane moist forest in Ailao Mountain and the tropical seasonal rainforest in Bubeng in this study. ALM, the subtropical montane moist forest in Ailao Mountains; BBV, the tropical seasonal rainforest in Bubeng. PF (primary forest), PFE (primary forest edge near the reservoir) and SF (secondary forest) are different habitats in ALM. LNC, leaf nitrogen concentrations; LPC, leaf phosphorus concentrations.

Uncovering patterns in leaf N-P scaling relationships is crucial for advancing our understanding of epiphyte adaptation and evolution. Our finding reveal distinct and consistent patterns in these relationships across spatial scales, underscoring the strong influence of the epiphytic habitat on the relative accumulation of N and P in leaves (**Figure 5**). This study provides new mechanistic insight by demonstrating that the epiphytic lifestyle fundamentally alters nutrient scaling relationships compared with terrestrial plants.

### Do epiphytes and terrestrial plants differ in β?

Vascular epiphytes exhibit distinct β compared with terrestrial plants. Global analysis reveals a higher β (0.78) for epiphytes than for terrestrial plants (Reich et al., 2010; Tian et al., 2018), indicating a faster relative accumulation of N with respect to P. Mechanistically, in SMA estimation β is proportional to the ratio of the dispersion of log-transformed N to that of log-transformed P (β ≈ SD[log N]/SD[log P]); therefore, greater variability in leaf N combined with relatively stable LPC mathematically yields a higher β (Warton et al., 2006; Guo et al., 2020). In epiphytes, the pronounced sensitively of leaf N to fluctuating N supply under arid or nutrient-poor conditions (Cheaib et al., 2025), together with high spatial–temporal heterogeneity in canopy nutrient inputs (Zotz, 2016), increases variability in leaf N. Meanwhile, epiphytes often exhibit “luxury consumption” of P, maintaining stable LPC despite environmental fluctuations (Winkler and Zotz, 2009; Hadi Alkarawi and Zotz, 2014; Zotz, 2016). Together, these factors explain the higher β observed in epiphytes. Lower N:P further support this interpretation. Our pooled data show that global epiphytes have a lower mean N:P ratio (11.09) than terrestrial plants (15.8, Tian et al., 2018), consistent with dominant N limitation in epiphytic habitats (Güsewell, 2004; Hietz et al., 2022; Liu et al., 2025). The lower N availability in epiphytic habitats may enhance the relative responsiveness of leaf N to nutrient inputs, producing faster proportional N accumulation with respect to P and thereby contributing to the higher β observed in epiphytes. This interpretation aligns with Guo et al. (2020), who proposed that N limitation influences N–P scaling primarily by altering relative investment patterns rather than by directly constraining leaf N concentrations. Supporting this, facultative epiphytes exhibit a lower β in epiphytic habitats (0.69) than in terrestrial habitats (0.91), indicating that when N becomes more available and stable—as in terrestrial environments—absolute N accumulation increases, but relative N variability decreases. Thus, nutrient availability regulates both the magnitude and proportionality of N and P accumulation, reconciling the apparent contrast between low- and high-N conditions.

Beyond nutrient limitation, several physiological and ecological characteristics of epiphytes may shape their leaf N-P scaling relationships. Because these plants rely primarily on intermittent atmospheric inputs rather than soil-derived nutrients (Zotz, 2016), their nutrient acquisition is closely linked to water balance (Querejeta et al., 2018). Morphological adaptations such as trichomes, velamen, and succulent tissues enhance the interception and retention of throughfall and mist, thereby influencing both total nutrient availability and within-tissue allocation (Zotz, 2016). Many epiphytes exhibit CAM or intermediate photosynthetic pathways that enhance water-use efficiency but reduce nutrient turnover (Gilman and Edwards, 2020; Zotz et al., 2021a). This constraint slows P cycling and results in a relative accumulation of N in structural and enzymatic compounds (Schleuss et al., 2020). Such physiological adjustments may explain the elevated β values observed, reflecting N’s dominant role in maintaining metabolic capacity under prolonged drought or fluctuating nutrient supply. In additions, the pronounced microclimatic heterogeneity of epiphytic habitats—characterized by large diurnal fluctuations in temperature and vapor pressure deficit—likely reinforce conservative nutrient-use strategies (Yu et al., 2023). Epiphytes exposed to higher irradiance and desiccation may allocate more N to photoprotective pigments and osmotic-regulating metabolites rather than growth-related functions (Adibah and Ainuddin, 2011; He et al., 2024; Liu et al., 2025). Collectively, these adaptive features suggest that the leaf N–P scaling relationship in epiphytes emerge from an integration of nutrient limitation, water balance, and photosynthetic physiology.

Notably, our study found no significant difference in β between vascular epiphytes and trees in the subtropical montane moist forest or the tropical seasonal rainforest. This similarity may result from two main factors. First, the leaf N–P scaling exponent of plant is likely constrained by macroscale climatic conditions (Tian et al., 2018; Guo et al., 2021). Consequently, both epiphytes and trees within the same forest type may experience similar climatic constraints, leading to convergent β values. Second, most vascular epiphytes in our dataset are herbaceous or fern species, whereas the terrestrial plants are mainly woody trees. In terrestrial ecosystems, herbaceous plants generally exhibit lower β values than woody species (Tian et al., 2018); however, the relatively higher β of epiphytes offsets this difference, resulting in no significant contrast between the epiphytic (herbaceous) and terrestrial (woody) groups. Future research should quantitatively assess the influence of climatic factors on β in epiphytes and compare the β of epiphytic herbaceous species with that of terrestrial herbaceous plants to better understand the combined effects of plant functional type and environment on nutrient-scaling relationships.

### Leaf N-P scaling exponents of epiphytes across different scales

Inconsistent with Tian et al. (2018), no significant differences in β values between epiphytic ferns and seed plants at both global and subtropical montane moist forest scales in our study. However, this supports the idea that β is uniform across major functional groups (Reich et al., 2010).

Additionally, no significant difference in β were observed among epiphytes across forest types. However, trees in the tropical seasonal rainforest exhibited a slightly lower β compared to those in the subtropical montane moist forest. This can be attributed to the tropical seasonal rainforest being more P-limited and warmer, which promotes faster P accumulation relative to N in trees, leading to a lower β (Tian et al., 2018a; Guo et al., 2020, 2021). In contrast, for epiphytes, the relative accumulation of N and P in leaves appears to be affected by N limitation in epiphytic habitats (**Figures 1**, **2**, **4**). Based on our analysis, epiphytes in these habitats tend to accumulate of N faster relative to P. Consequently, the differences in β between epiphytes in the two forest types are less pronounced than those observed in trees.

At the local scale, β values showed no significant variation across habitats. A previous study in the subtropical montane moist forest of the Ailao Mountains, Southwest China, found that epiphytes in primary forests exhibited higher annual rhizome growth than those in secondary forests (Chen et al., 2019), suggesting faster growth rates and greater P accumulation in primary forests (Guo et al., 2020). Yet, epiphytes in primary forests did not display a lower β compared to those in secondary forests. Combined with our findings, this suggests that the unique characteristics of epiphytic habitats—particularly their reliance on atmospheric nutrient inputs (e.g., through rain and canopy deposition) and detrital canopy soils, coupled with limited access to groundwater (Zotz, 2016)—constrain leaf N-P scaling relationship, favoring a trade-off in nutrient uptake that leans more towards N than P (Guo et al., 2020).

Interestingly, we observed a decreasing trend in the correlation between leaf N and P concentrations from primary forests adjacent to the reservoir to primary forests and secondary forests. This aligns with the view that changes in global water availability can decouple plant N:P (Yuan and Chen, 2015). To cope with limited water supply, a significant fraction of N in epiphytes is allocated to functions other than growth, such as water retention and stress tolerance (Sardans et al., 2012; Zotz, 2016). This allocation likely contributes to the weaker correlation between LNC and LPC in secondary forests.

While this study offers new insights into the mechanisms governing N–P scaling relationships in vascular epiphytes, several limitations should be noted. First, field sampling was confined to two forest types in southwest China, which may not capture spatial variation across broader biogeographic gradients. Second, the taxonomic coverage of epiphytes was uneven, with some functional groups and lineages underrepresented. Moreover, we did not directly measure microclimatic or physiological variables—such as canopy humidity, nutrient uptake rates, or photosynthetic efficiency—that could further elucidate the drivers of N–P coupling. Future work integrating trait-based experiments, and nutrient manipulation studies will help refine these conclusions and strengthen causal inference.

## Conclusions

In this study, we investigated the leaf N-P scaling exponent of vascular epiphytes in comparison to terrestrial plants, examined its variation across different scales (**Figure 5**). Based on our findings, we conclude that (1) global vascular epiphytes exhibit distinct leaf N-P scaling exponents compared to terrestrial plants; (2) there are no significant differences in β across functional groups, forest types, or habitats, indicating the constraints imposed by the epiphytic lifestyle. Overall, this study uncovers key patterns in the leaf N-P scaling relationships of vascular epiphytes, and emphasizes the critical role of the epiphytic habitat in shaping these relationships, thereby advancing our understanding of epiphyte adaptation and evolution.

## Data Availability

The original contributions presented in the study are included in the article/**Supplementary Material**. Further inquiries can be directed to the corresponding author.
