# Supplementary material for: The scaling relationship between leaf nitrogen and phosphorus concentrations in vascular epiphytes
Source: Front Plant Sci. 2026 Jan 2;16:1712082. doi: 10.3389/fpls.2025.1712082 (PMC12808357; doi:10.3389/fpls.2025.1712082)
Supplement: Supplementary file 3 [file Table3.docx]

**Table S1.** Summary of SMA regression results between leaf N and P concentrations. The data of global terrestrial plants was from Tian et al (2018). TSR, tropical seasonal rainforest; SMF, subtropical montane forest; LNC, leaf nitrogen concentration; LPC, leaf phosphorus concentration.

|  | Group | *N* | β (95% CI) | R^2^ | LNC (g·kg^-1^) | LPC (g·kg^-1^) | N:P |
| --- | --- | --- | --- | --- | --- | --- | --- |
| **Global** |  |  |  |  |  |  |  |
|  | Epiphytic individuals | 1803 | 0.78[0.75, 0.81] | 0.36 | 14.71 | 1.65 | 11.09 |
|  | Terrestrial plants | 12055 | 0.68[0.67, 0.69] | 0.33 | 19.93 | 1.20 | 15.8 |
|  | Epiphytic species | 323 | 0.69[0.64, 0.75] | 0.45 | 14.54 | 1.48 | 12.18 |
|  | Epiphytic ferns | 103 | 0.65[0.55, 0.76] | 0.29 | 16.09 | 1.49 | 13.10 |
|  | Epiphytic seed plants | 220 | 0.69[0.63, 0.76] | 0.51 | 13.81 | 1.47 | 11.75 |
| **Local** |  |  |  |  |  |  |  |
| TSR | Epiphytes | 19 | 0.67[0.50, 0.90] | 0.65 | 13.43 | 1.43 | 11.05 |
|  | Trees | 18 | 0.60[0.41, 0.88] | 0.45 | 19.15 | 1.32 | 15.69 |
| SMF | Epiphytes | 20 | 0.84[0.60, 1.18] | 0.50 | 21.47 | 2.07 | 10.92 |
|  | Trees | 16 | 1.01[0.67, 1.53] | 0.46 | 16.88 | 1.14 | 15.17 |
|  | Epiphytic ferns | 219 | 0.73[0.65, 0.82] | 0.26 | 22.54 | 2.27 | 11.29 |
|  | Epiphytic seed plants | 124 | 0.76[0.65, 0.82] | 0.59 | 20.31 | 2.15 | 11.06 |
|  | Epiphytes in PFE | 107 | 0.72[0.63, 0.81] | 0.62 | 22.14 | 2.16 | 12.11 |
|  | Epiphytes in PF | 161 | 0.80[0.71, 0.91] | 0.34 | 21.42 | 2.17 | 11.07 |
|  | Epiphytes in SF | 75 | 0.76[0.62, 0.93] | 0.22 | 21.82 | 2.43 | 10.21 |
| Facultative  epiphytes | Epiphytic individuals | 39 | 0.69[0.58, 0.82] | 0.71 | 27.15 | 2.30 | 10.11 |
|  | Terrestrial individuals | 43 | 0.91[0.80, 1.04] | 0.84 | 25.52 | 2.19 | 11.78 |
